# Clinical Epidemiology of Reduced Kidney Function among Elderly Male Fishing and Agricultural Population in Taipei, Taiwan

**DOI:** 10.1155/2013/214128

**Published:** 2013-11-13

**Authors:** Chi-Mei Kuo, Wu-Hsiung Chien, Hsi-Che Shen, Yi-Chun Hu, Yu-Fen Chen, Tao-Hsin Tung

**Affiliations:** ^1^School of Medicine, Tzu-Chi University, Hualien 970, Taiwan; ^2^Department of Family and Community Medicine, Cheng-Hsin General Hospital, Taipei 112, Taiwan; ^3^New Taipei City Hospital, Taipei 241, Taiwan; ^4^Taipei Medical University, Taipei 110, Taiwan; ^5^Department of Healthcare Management, Yuanpei University, Hsinchu 300, Taiwan; ^6^Oriental Institute of Technology, Taipei 220, Taiwan; ^7^NHI Dispute Mediation Committee, Ministry of Health and Welfare, Taipei 103, Taiwan; ^8^Institute of Health and Welfare Policy, National Yang-Ming University, Taipei 112, Taiwan; ^9^Department of Nursing, Kang-Ning Junior College of Medical Care and Management, Taipei 114, Taiwan; ^10^School of Medicine, Faculty of Public Health, Fu-Jen Catholic University, New Taipei City 242, Taiwan; ^11^Department of Medical Research and Education, Cheng-Hsin General Hospital, Shih-Pai, Taipei 112, Taiwan; ^12^Department of Crime Prevention and Correction, Central Police University, Taoyuan 333, Taiwan

## Abstract

*Purpose*. To quantify the prevalence of and associated factors for chronic kidney disease (CKD) among male elderly fishing and agricultural population in Taipei, Taiwan. *Methods*. Subjects (*n* = 2,766) aged 65 years and over voluntarily admitted to a teaching hospital for a physical checkup were collected in 2010. CKD was defined as an estimated glomerular filtration rate <60 mL/min/1.73 m^2^. *Results*. Among these subjects, the over prevalence of chronic kidney disease was 13.6% (95% CI: 12.3–14.9%). The age-specific prevalence of CKD in 65–74 years, 75–84 years, and ≥85 years was 8.2%, 19.1%, and 27.0%, respectively. From the multiple logistic regression, age (OR = 1.05, 95% CI: 1.02–1.09), hyperuricemia (OR = 2.94, 95% CI: 1.90–3.78), central obesity (OR = 1.17, 95% CI: 1.02–1.56), hyperglycemia (OR = 1.23, 95% CI: 1.11–1.67), hypertriglyceridemia (OR = 1.25, 95% CI: 1.08–1.66), and lower HDL-C (OR = 1.61, 95% CI: 1.23–1.92) were statistically significantly related to CKD. The presence of metabolic components (one or two versus none, OR = 1.10, 95% CI: 1.04–1.25; three or more versus none, OR = 2.12, 95% CI: 1.86–2.78) also appeared to be statistically significantly related to CKD after adjustment for other independent factors. *Conclusion*. Several clinical factors independently affect the development of CKD in the elderly male fishing and agricultural population.

## 1. Introduction

 The prevalence of chronic kidney disease (CKD) is increasing rapidly worldwide and is now recognized as a global public health problem [1]. CKD can be divided into five stages based on the appearance of proteinuria and glomerular filtration rate (GFR) levels. To estimate GFR, the modification of diet in renal disease (MDRD) formula was recommended for its accuracy in elderly subjects [2]. A better understanding of the etiology of CKD, leading to early detection and prevention and effective therapy might alleviate the future burden of end-stage renal disease (ESRD), cardiovascular disease (CVD), and its associated mortality [3].

CKD is matched the Wilson criteria for screening due to it is an important health problem; the disease natural history should be understood; a recognizable latent or early symptomatic stage; a test is easy to perform and interpret, acceptable, accurate, reliable, sensitive and specific; an accepted treatment recognized for the disease; treatment is more effective if started early; a policy on who should be treated; diagnosis and treatment are cost-effective; and case-finding should be a continuous process [4]. Undoubtedly, the requirements of good health and appropriate training for agricultural and fishing population are necessary. The long hours or irregular working hours may cause some adverse health effects. To the best of our knowledge, however, few clinical evidence-based studies attempted to determine the prevalence and possible etiology of CKD for the elderly male agricultural and fishing population of Taiwan, which also faced the burden of this disorder. In order to identify the prevalence of and the associated risk factors for CKD, this study was designed to explore the potential for condition-related factors, because it was considered to know underscore important implications for the understanding of the overall pathogenesis of CKD in this subelderly population. As mentioned above, the purpose of this study is to explore the context of prevalence of and cardiovascular risk factors for CKD amongst the elderly male agricultural and fishing population, as determined by the application of a healthy volunteer subjects screening program health examination in Taipei, Taiwan.

## 2. Methods

### 2.1. Study Design and Data Collection

This cross-sectional study was conducted with a total of 2,766 male aged 65 years and over healthy occupational adults with agricultural and fishing professional fields voluntarily admitted to one teaching hospital in Northern Taiwan for an annual physical checkup between January 1, 2010 and December 31, 2010. All procedures were performed in accordance with the guidelines of our institutional ethics committee and adhered to the tenets of the Declaration of Helsinki. All patients' information was anonymous.

The medical histories and measurements of the participants were obtained by well-trained nurses. Personal and family histories of hypertension, type 2 diabetes, cardiovascular diseases, and other chronic diseases were obtained by a structured health interview questionnaire. The study participants were asked to take off the shoes and any other belongings that could possibly add extra weight when they were weighed. Heights and weights were evaluated according to body mass index (BMI). Also the waist circumference was also measured at the level of the iliac processes and the umbilicus with a soft tape measure to estimate abdominal obesity. Blood pressures for each subject were measured twice in the sitting position with an interval of 15 minutes between the measurements, by means of standard sphygmomanometers of appropriate width, after a rest period for 30 minutes. Those who are taking antihypertensive therapy were considered to be known hypertension.

Fasting blood samples were drawn via venipuncture from study participants by clinical nurses. Overnight-fasting serum and plasma samples (from whole blood preserved with EDTA and NaF) were kept frozen (−20°C) until they are ready for analysis. 

The estimated glomerular filtration rate (eGFR) was calculated using the MDRD equation, which was modified for data from Chinese CKD patients [1, 5]. The reduced renal function was defined as an eGFR < 60 mL/min/1.73 m^2^: eGFR (mL/min/1.73 m^2^) = 175 × calibrated serum creatinine (mg/dL)^−1.234^  ×  age (year)^−0.179^. 

Metabolic syndrome was diagnosed according to NCEP ATP III criteria, that is, at least 3 of the following 5 parameters should be present: abdominal obesity (waist circumference > 90 cm for males), hypertension (SBP > 130 mm Hg and/or DBP > 85 mm Hg) or history of antihypertensive usage, hypertriglyceridemia (≥150 mg/dL) or presence of treatment for this disorder, low HDL-C (<40 mg/dL in males) or presence of treatment for this disorder, and high fasting plasma glucose (>100 mg/dL) or presence of diagnosis of type 2 diabetes [6, 7]. 

For the health behavior factors, participants were asked in multiple choice formats to describe their intake of alcohol drinks. Bear, wine, and spirits were assessed separately. Current alcohol consumption was assessed by the question “How many cups, glasses, or drinks of these beverages do you usually drink a day or a month, and for how many years?” People who reported drinking were classified on the basis of the sum of their reported current consumption of all types of alcoholic beverages. We categorized daily ethanol intake in grams into 4 categories: nondrinkers, <20 g (mild), 20–70 g (moderate), and >70 g (heavy). Nondrinkers are defined as the people who explicitly recorded zero for current alcohol consumption of any alcoholic beverage and zero or blank for previous consumption. Heavy drinker is denied as daily alcohol intake >70 g [8]. Smoking habits were classified according to their current smoking status into 3 groups: those who have never smoked, ex-cigarette smokers, and current smokers. Current smoking is defined as the use of at least 1 cigarette per day during the preceding years [9]. In addition, two groups were defined for physical exercise, that is, three or more times per week and less than three times.

## 3. Statistical Analysis

Statistical analysis was performed using SPSS for Windows, (SPSS version 18.0; Chicago, IL, USA). The two-sample independent *t*-test and one-way ANOVA method were adopted to assess differences in the mean value of continuous variables. The *χ* ^2^-trend test was used to determine significant differences in proportions among categorical variables. Mantel-Haenszel statistics are used in the analysis of stratified categorical data. Multiple logistic regression was also performed to investigate the independence of factors associated with the prevalence of CKD. A *P* value of < 0.05 was considered to represent a statistically significant difference between two test populations.

## 4. Results

**Figure 1:**
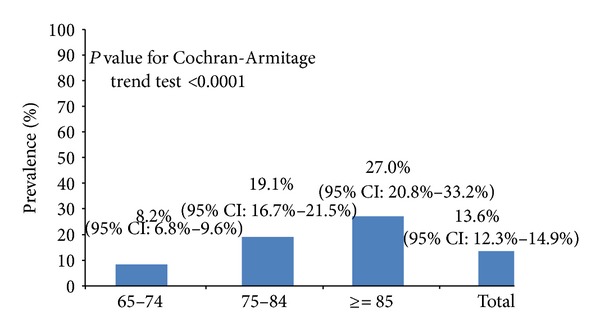
Age-specific prevalence of chronic kidney disease among elderly male fishing and agricultural population (*n* = 2, 766).

As Figure 1 shows, the overall prevalence of CKD for the study participants was 13.6% (95% CI: 12.3%–14.9%). From the Cochran-Armitage trend test, the prevalence of each type of CKD showed an increase with age (*P* < 0.0001). Subjects aged 85 years and over (27.0%, 95% CI: 20.8%–33.2%) had more than 3-fold risk for CKD compared with the subjects aged 65–74 years (8.2%, 95% CI: 6.8%–9.6%).

Table 1 shows the demographic characteristics of the participants who were and were not diagnosed with CKD. In addition to DBP, waist circumference, fasting blood glucose, triglycerides, HDL-C, uric acid, and ALT were significantly different in age subgroups. Using the two-sample independent *t*-test, the associated factors that were significantly related to CKD included metabolic component, BMI, serum uric acid, and ALT.

The relationship proportion of Chinese elderly male with CKD and individual components is shown in Table 2. Elevated blood pressure, central obesity, hyperglycemia, hypertriglyceridemia, and low HDL-C were statistically significantly associated with an increased age-specific prevalence of CKD. There was a significant dose-response relationship between the number of metabolic syndrome components and the prevalence of CKD.

The effect of independent associated risk factors on CKD was examined using the multiple logistic regression model. As is depicted in Table 3, subsequent to adjustment for confounding factors, age (OR = 1.05, 95% CI: 1.02–1.09), hyperuricemia (yes versus no, OR = 2.94, 95% CI: 1.90–3.78), central obesity (yes versus no, OR = 1.17, 95% CI: 1.02–1.56), hyperglycemia (yes versus no, OR = 1.23, 95% CI: 1.11–1.67), hypertriglyceridemia (yes versus no, OR = 1.25, 95% CI: 1.08–1.66), and lower HDL-C (yes versus no, OR = 1.61, 95% CI: 1.23–1.92). The presence of metabolic components (one or two versus none, OR = 1.10, 95% CI: 1.04–1.25; three or more versus none, OR = 2.12, 95% CI: 1.86–2.78) also appeared to be statistically significantly related to CKD after adjustment for other independent factors.

## 5. Discussion

### 5.1. Prevalence and Cardiovascular Factors for the Development of Chronic Kidney Disease

All GFR estimating equations have some limitations due to diet or clinical conditions such as malnutrition and inflammation may also affect the applicability of the MDRD study equation for use with Asians, while the Cockcroft-Gault equation tends to overestimate the true GFR [10]. Older age represented significant risk factors related to the likelihood of a CKD after adjustment for confounding factors in this study. A previous study also indicated the prevalence of CKD with age in males, particularly those at 60 years of age and over [11]. Renal functions deteriorate in the aged population for various reasons. The subjects might have had a renal disease, such as nephrosclerosis or ischemic kidney disease which partly explain the higher prevalence of CKD in the older population [11]. However, serum creatinine is correlated with muscle mass and therefore the evaluation of eGFR using MDRD equation in subjects with extremes of muscle mass is subject to inaccuracy. In those with increased muscle mass, eGFR will be underestimated, whereas in those with reduced muscle mass, eGFR will be overestimated [12, 13]. The MDRD formula is accurate in patients with CKD, but it significantly underestimates eGFR in healthy people due to the exclusion of healthy people from the study used to develop this equation [13]. This agricultural and fishing study population was engaged in high physical activity and likely had more muscle mass compared to general population which could make the eGFR falsely lower. This implied that the classification of CKD might be confounded.

The most common types of morphological renal lesions observed in renal biopsies of obese patients are mainly focal and segmental glomerulosclerosis and glomerulomegaly [14]. Previous epidemiological studies indicated that obesity is associated with CKD [15–17]. However, few studies examined the relationship between body fat distribution (central versus peripheral adiposity) and the risk for CKD, even though central adiposity is a better predictor of hypertension, dyslipidemia, and the metabolic syndrome than BMI alone [15, 18]. In this study, we found that the central obesity but not higher BMI was significantly related to CKD. The mechanisms of obesity-induced renal injury are not fully understood and are likely to involve a combination of hemodynamic and metabolic abnormalities [14]. The rapid and parallel increases in the prevalence of ESRD and obesity suggested that obesity may be a major risk factor for kidney disease through other mechanisms than diabetes and hypertension [14].

Metabolic syndrome is more prevalent among males and the incidence increases with age [10]. Consistent with previous studies [3, 11, 14, 19–22], our results show that the metabolic syndrome is strongly associated with CKD. This suggests that the level of metabolic syndrome is an indicator for the deterioration of CKD. By fitting data with a logistic regression model, the odds ratios for the metabolic syndrome in the three categories were estimated as 1.15 (95% CI: 1.06–1.27) in the one or two metabolic components and 2.22 (95% CI: 1.91–2.64) in the three or more metabolic components, as compared with the no metabolic components group. This finding suggests that the onset of CKD may begin up to the subject with metabolic risk factors. In addition, there are multiple mechanisms of CKD among metabolic components, and these are not yet well delineated [10, 23]. Hypertriglyceridemia and increased adipocytokine levels are possible pathophysiologic mechanisms for metabolic syndrome [24]. Excessively secreted plasminogen activator inhibitor type 1 (PAI-1) might also affect each component of metabolic syndrome [25]. The greater the number of metabolic components indicated, the greater the prevalence and incidence of CKD [10]. Previous study also found that GFR decreased significantly faster in patients with four or more metabolic syndrome components compared with those who had one or no components [20]. From the preventive medicine viewpoint, targeted screening should be directed at subgroups of the population that would derive the most benefit from CKD detection such as those with metabolic syndrome [10].

Each component of metabolic syndrome, per se, may cause renal function damage [10]. We found that hyperglycemia, hypertriglyceridemia, and lower HDL-C were significantly associated with a higher risk of CKD after being controlled for confounding factors. Epidemiologic studies showed that diabetes is the major risk factor for the development and progression of CKD [6]. CKD also may be considered as an additional complication of macrovascular atherosclerosis during prediabetes [26]. In addition, dyslipidemia is related to the progression of CKD. Inflammatory cytokines that are produced by adipose tissue, the adipocytokines, have a role in renal damage in patients with metabolic syndrome [10, 27]. However, our results indicated that elevated blood pressure showed no clear association with CKD. Other studies in Asia revealed the similar results but this is not the same in the western countries [3, 6, 28, 29]. The disparity may be explained by race or ethnic differences. 

In this study, hyperuricemia exhibited a significant high OR value and was therefore one of the strongest risk factors related to CKD. Other studies also showed that hyperuricemia is not only associated with CKD, but also independent of the presence of metabolic syndrome [1, 22]. Hyperuricemia has been found to accelerate renal disease in the remnant kidney model and to accelerate experimental cyclosporine nephropathy [30–32]. This implied that hyperuricemia may be a direct pathogenic factor in CKD [1, 33]. However, the effect of this clinical serum marker on progression on kidney disease in humans remains unclear [33]. 

### 5.2. Perceived Limitations

One of the major limitations to this study population is selected on a voluntary basis based on one area elderly population screened, which would potentially introduce selection bias. Voluntary bias can be defined as that comes from the fact that a particular sample can contain only those participants who are actually willing to participate in the study and who participate and find the topic particularly interesting and who are more likely to volunteer for that study, with the same being for those who are expected to be evaluated on a positive level [34]. The potential influence on the prevalence estimated and the study-observed CKD-associated risk factors were inevitable. Nevertheless, we still retained sufficient statistical power to be able to effectively evaluate the various associated risk factors for CKD subsequent to adjustment for the found factors given the relative large sample size. Secondly, this study only included subjects who were aged ≥65 years and may have different characteristics compared with the whole male population. However, this subpopulation was more susceptible to have CKD and easily to know the trend happened in Taiwan and take early prevention strategies. Finally, our measurements were conducted at only a single point in time and, by clear inference, would not be able to be used to reflect long-term exposure to various demographic or biochemical aspects or factors, which might be important influencers of CKD. The solution to such a quandary would best be accomplished by conducting a number of prospective longitudinal analogous studies, the results of which would be expected to complement the cross-sectional findings of this study.

## 6. Conclusion

The prevalence of CKD is related to older age, hyperuricemia, central obesity, hyperglycemia, hypertriglyceridemia, lower HDL-C, and metabolic syndrome in this study. Further studies are needed to elucidate the temporal sequence of events that typically lead to CKD among elderly population. In order to prevent the CKD, promoting this population with controlled obesity, uric acid, and health improvement for metabolic function is important.

## Figures and Tables

**Table 1:** Demographic characteristics of participants with and without chronic kidney disease (*n* = 2,766).

Variables	General (*n* = 2766)	Age				Chronic kidney disease		
		65–74 (*n* = 1537)	75–84 (*n* = 1033)	≥85 (*n* = 196)	*P* value for *F*-test	Yes (*n* = 376)	No (*n* = 2390)	*P* value for *t*-test
	mean ± SD	mean ± SD	mean ± SD	mean ± SD		mean ± SD	mean ± SD	
Age (year)	74.4 ± 6.6	—	—	—	—	77.7 ± 6.8	73.9 ± 6.4	<0.001
SBP (mm Hg)	137.7 ± 35.8	137.3 ± 43.6	138.1 ± 22.3	138.6 ± 22.5	0.78	142.8 ± 54.0	136.9 ± 31.9	0.003
DBP (mm Hg)	79.1 ± 21.5	81.4 ± 26.5	76.6 ± 12.0	73.9 ± 12.8	<0.001	79.6 ± 28.5	79.0 ± 20.2	0.67
BMI (kg/m²)	27.2 ± 73.9	27.4 ± 66.9	27.5 ± 89.3	23.6 ± 3.9	0.78	25.5 ± 4.1	27.4 ± 79.5	0.64
Waist circumference (cm)	88.8 ± 17.2	89.1 ± 10.1	88.8 ± 24.8	85.7 ± 11.7	0.03	90.8 ± 9.7	88.5 ± 18.1	0.02
Fasting blood glucose (mg/dL)	99.6 ± 27.3	100.7 ± 29.0	98.8 ± 26.0	95.8 ± 18.8	<0.001	100.9 ± 22.8	99.4 ± 27.9	0.33
Triglycerides (mg/dL)	130.0 ± 90.8	137.9 ± 101.6	121.7 ± 77.9	111.7 ± 50.0	<0.001	148.0 ± 92.0	127.1 ± 90.4	<0.001
Total cholesterol (mg/dL)	195.4 ± 33.6	199.0 ± 33.7	191.5 ± 32.8	187.5 ± 33.4	0.90	192.5 ± 35.7	195.8 ± 33.2	0.72
HDL-C (mg/dL)	51.8 ± 14.4	51.9 ± 14.6	51.7 ± 14.3	52.0 ± 13.5	0.03	47.4 ± 13.6	52.5 ± 14.4	<0.001
Uric acid (mg/dL)	6.5 ± 1.5	6.4 ± 1.5	6.5 ± 1.5	6.6 ± 1.4	<0.001	7.6 ± 1.8	6.3 ± 1.4	<0.001
ALT (U/L)	32.4 ± 25.8	34.5 ± 32.1	30.5 ± 14.5	20.1 ± 9.4	0.03	30.1 ± 16.0	32.7 ± 27.0	0.07

**Table 2:** The relationship between metabolic components and chronic kidney disease in the study participants (*n* = 2,766).

	65–74 yrs	75–84 yrs	≥85 yrs	Total	*P* value for Mantel-Haenszel *χ* ^2^ test
	CKD Prevalence (95% CI)	CKD Prevalence (95% CI)	CKD Prevalence (95% CI)	CKD Prevalence (95% CI)	
Metabolic components					
Elevated blood pressure	9.1 (7.3–10.9)	19.4 (16.5–22.4)	33.3 (25.2–41.4)	14.8 (13.2–16.4)	0.02
Central obesity	11.0 (8.8–13.3)	22.4 (18.7–26.1)	32.9 (22.1–43.7)	16.5 (14.5–18.5)	<0.001
Hyperglycemia	12.3 (9.4–15.2)	23.1 (18.5–27.7)	31.9 (18.6–45.2)	17.4 (14.9–19.9)	<0.001
Hypertriglyceridemia	13.2 (10.1–16.3)	27.2 (21.5–32.9)	30.6 (15.6–45.7)	18.6 (15.8–21.4)	<0.001
Low HDL-C	16.0 (12.1–19.9)	28.1 (22.2–34.0)	35.7 (21.2–50.2)	21.8 (18.5–25.1)	<0.001
Number of components of metabolic syndrome					
None	3.0 (0.8–5.2)	5.9 (4.3–7.5)	14.2 (11.2–17.2)	8.5 (5.8–11.2)	
One or two	16.4 (10.4–22.4)	15.1 (12.2–18.0)	27.6 (22.6–32.6)	11.1 (9.5–12.7)	<0.001
Three or more	12.1 (1.0–23.2)	27.2 (19.0–35.4)	36.7 (23.2–50.2)	20.3 (17.6–23.0)	

**Table 3:** Multiple logistic regression on the risk factors associated with the chronic kidney disease among male elderly fishing and agricultural population (*n* = 2, 766).

Variables	CKD versus non-CKD		
	Odds ratio	95% Confidence interval	*P* value
Model A			
Age (year)	1.05	1.02–1.09	0.001
Smoking (yes versus no)	0.87	0.66–1.18	0.32
(ex-smoker versus no)	0.93	0.71–1.16	0.40
Alcohol drinking (<20 g/day versus no)	1.03	0.81–1.39	0.26
(20–70 g/day versus no)	0.88	0.69–1.12	0.14
(≥70 g/day versus no)	1.20	0.92–1.47	0.07
Physical exercise (≥3 versus <3 times/week)	0.79	0.62–1.04	0.06
Hyperuricemia (yes versus no)	2.94	1.90–3.78	<0.001
Central obesity (yes versus no)	1.17	1.02–1.56	0.03
Elevated blood pressure (yes versus no)	1.07	0.81–1.42	0.37
Hyperglycemia (yes versus no)	1.23	1.11–1.67	0.01
Hypertriglyceridemia (yes versus no)	1.25	1.08–1.66	0.01
Lower HDL-C (yes versus no)	1.61	1.23–1.92	0.02
Obesity (yes versus no)	1.05	0.87–1.31	0.28
Higher ALT (yes versus no)	1.03	0.73–1.35	0.16

Model B			
Age (year)	1.03	1.01–1.08	0.03
Smoking (yes versus no)	0.89	0.63–1.21	0.37
(ex-smoker versus no)	0.91	0.74–1.15	0.44
Alcohol drinking (<20 g/day versus no)	1.01	0.78–1.43	0.35
(20–70 g/day versus no)	0.86	0.65–1.14	0.19
(≥70 g/day versus no)	1.11	0.84–1.40	0.09
Physical exercise (≥3 versus <3 times/week)	0.84	0.65–1.03	0.11
Hyperuricemia (yes versus no)	2.15	1.57–2.99	<0.001
Metabolic components			
(One or two versus none)	1.10	1.04–1.25	0.04
(Three or more versus none)	2.12	1.86–2.78	<0.001
Obesity (yes versus no)	1.04	0.82–1.39	0.27
Higher ALT (yes versus no)	1.04	0.71–1.32	0.19
